# Role of Environmental Factors in Shaping Spatial Distribution of *Salmonella enterica* Serovar Typhi, Fiji

**DOI:** 10.3201/eid2402.170704

**Published:** 2018-02

**Authors:** Ruklanthi de Alwis, Conall Watson, Birgit Nikolay, John H. Lowry, Nga Tran Vu Thieu, Tan Trinh Van, Dung Tran Thi Ngoc, Kitione Rawalai, Mere Taufa, Jerimaia Coriakula, Colleen L. Lau, Eric J. Nilles, W. John Edmunds, Mike Kama, Stephen Baker, Jorge Cano

**Affiliations:** London School of Hygiene and Tropical Medicine, London, UK (R. de Alwis, C. Watson, B. Nikolay, W.J. Edmunds, J. Cano);; Oxford University Clinical Research Unit, Ho Chi Minh City, Vietnam (R. de Alwis, N.T.V. Thieu, T.T. Van, D.T.T. Ngoc, S. Baker);; Oxford University, Oxford, UK (R. de Alwis, S. Baker);; University of the South Pacific, Suva, Fiji (J.H. Lowry);; Project Heaven, Suva (K. Rawalai);; Ministry of Health and Medical Services, Suva (M. Taufa, M. Kama);; Fiji National University, Suva (J. Coriakula);; Australian National University, Canberra, Australian Capital Territory, Australia (C.L. Lau);; World Health Organization Western Pacific Region, Suva (E.J. Nilles);; Brigham and Women’s Hospital, Boston, Massachusetts, USA (E.J. Nilles);; Harvard Medical School, Boston (E.J. Nilles)

**Keywords:** Salmonella enterica serovar Typhi, bacteria, typhoid fever, environmental factors, risk factors, multilevel analysis, flooding, Vi capsular antigen, Vi antibodies, seroprevalence, Fiji

## Abstract

Fiji recently experienced a sharp increase in reported typhoid fever cases. To investigate geographic distribution and environmental risk factors associated with *Salmonella enterica* serovar Typhi infection, we conducted a cross-sectional cluster survey with associated serologic testing for Vi capsular antigen–specific antibodies (a marker for exposure to *Salmonella* Typhi in Fiji in 2013. Hotspots with high seroprevalence of Vi-specific antibodies were identified in northeastern mainland Fiji. Risk for Vi seropositivity increased with increased annual rainfall (odds ratio [OR] 1.26/quintile increase, 95% CI 1.12–1.42), and decreased with increased distance from major rivers and creeks (OR 0.89/km increase, 95% CI 0.80–0.99) and distance to modeled flood-risk areas (OR 0.80/quintile increase, 95% CI 0.69–0.92) after being adjusted for age, typhoid fever vaccination, and home toilet type. Risk for exposure to *Salmonella* Typhi and its spatial distribution in Fiji are driven by environmental factors. Our findings can directly affect typhoid fever control efforts in Fiji.

With an estimated disease burden of 20.6 million cases in low- and middle-income countries in 2010, typhoid fever remains an enteric disease of public health concern (*1*,*2*). Typhoid fever cases largely arise in low- and middle-income countries because marked improvements in water, sanitation, and sewage removal have helped reduce typhoid fever incidence in most industrialized countries (*3*–*6*). *Salmonella enterica* serovar Typhi, the causative agent of typhoid fever, is specific to humans and is typically transmitted by the fecal–oral route between humans, that is, through the ingestion of contaminated food and water (*3*,*7*). Typhoid fever infections are usually acute, although for ≈3%–5% of cases, *Salmonella* Typhi causes an asymptomatic and persistent (chronic) infection. These infected persons are commonly referred to as typhoid fever carriers and are capable of shedding bacteria and sustaining transmission within the community (*3*,*8*).

Pathogenicity of *Salmonella* Typhi is conferred by virulence factors, such as Vi polysaccharide. The Vi polysaccharide is an outer capsular antigen that enables greater human infectivity than those *Salmonella* Typhi strains not expressing the antigen (*9*). Because of the highly antigenic nature of Vi, infection with Vi-positive *Salmonella* Typhi strains elicits Vi-specific antibodies in humans (*10*). Therefore, detection of Vi-specific IgG can be used to measure *Salmonella* Typhi exposure, either past or chronic infection(s) (*11*). Furthermore, current human-approved typhoid fever vaccines are primarily Vi antigen based (e.g., Vi polysaccharide and Vi conjugate vaccines) (*12*). Despite antigenicity of the Vi polysaccharide, antibodies and immunity conferred by the Vi vaccine are short lived (*13*).

Fiji is an archipelago of >300 islands in the Pacific Ocean; most of its population is on the 2 islands of Viti Levu and Vanua Levu. During 1991–2000, <5 typhoid cases/100,000 persons were reported per year, mostly in Vanua Levu (*14*,*15*). However, since 2005, the number of typhoid fever cases has been increasing (*16*), reaching a peak of >50 cases/100,000 persons/year after widespread destruction and flooding caused by Cyclone Tomas in 2010. As a result, the Fiji Ministry of Health increased surveillance and implemented additional prevention strategies, such as vaccination against typhoid fever in the worst affected regions (*17*,*18*).

The risk factors for transmission of *Salmonella* Typhi in Fiji are only partially understood. Inadequate handwashing practices, poor sanitation, lack of access to safe water, and dumping of untreated waste/sewage are believed to contribute to this transmission (*17*,*19*). In addition, every year during November–April, Fiji experiences powerful cyclones, which have led to destruction of homes and contamination of water sources by extensive rainfall and flooding, followed by an increase in diarrheal diseases (*20*,*21*). Although flooding has been shown to lead to outbreaks of other foodborne and waterborne diseases (*22*–*24*), a direct link between flooding and increased typhoid fever incidence has not been confirmed in Fiji.

Public health efforts to control typhoid fever have been hampered by the lack of information regarding the epidemiology, spatial distribution, and risk factors for typhoid fever exposure in Fiji. Therefore, we used the presence of Vi-specific antibodies as a biomarker for typhoid fever exposure and used geospatial and statistical approaches to identify environment-associated risk factors in the general population of Fiji. Because of the yearly occurrence of cyclones in Fiji, we gave special attention to the potential contribution of flooding (and flood-promoting factors) to seropositivity of *Salmonella* Typhi Vi antigen.

## Methods

### Study Design

This study was a cross-sectional cluster survey with an associated serologic analysis, which was conducted across 3 divisions in Fiji: Northern, Central, and Western. We excluded administrative areas where the 2010 typhoid vaccination campaign (*18*) had been implemented. We divided the country into healthcare coverage areas (nursing zones) and selected them by using probability proportional-to-size random sampling based on census data. We then selected cluster sites (communities) within nursing zones by using random list sampling, followed by random sampling of households within community cluster sites by using community health worker censuses or a modified Expanded Program on Immunization sampling of the World Health Organization (*25*) and then random sampling of a person per household. We excluded children <1 year of age. Community visits and data collection took place during September–December 2013, and entailed questionnaire administration, blood sample collection, and geolocation of surveyed households. Geographic coordinates were collected by using handheld geographic positioning system (GPS) devices at the house of a participant or the nearest community center. We calculated sample size at α = 0.05 by using expected seroprevalence informed from a previous study (*26*). Further details on study design and sampling have been reported by Watson et al. (*26*).

Informed consent was obtained in writing or by thumb print from all adult participants and parents or guardians of participating children. Written consent was obtained from children >12 years of age. This study was approved by the Fiji National Research Ethics Review Committee (#201303) and the London School of Hygiene and Tropical Medicine Ethics Committees (#6344 and #9187).

### Survey Data

**Figure 1:**
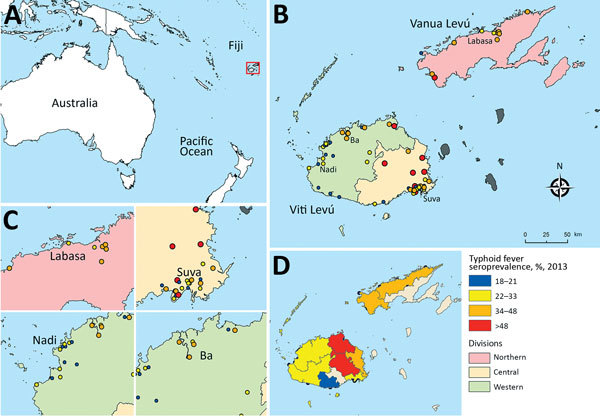
Geographic distribution of antibodies against Vi capsular antigen of *Salmonella enterica* serovar Typhi, Fiji, 2013. A) Location of Fiji islands in the southern Pacific Ocean. B) Seroprevalence of Vi antibody in sampled communities in 2013. C) Details of typhoid seroprevalence in large cities in Fiji (Labasa, Suva, Nadi, and Ba). D) Typhoid seroprevalence estimated for subdivisions in Fiji.

Information for 44 variables was collected during the cross-sectional survey as described (*26*). We then selected 13 survey variables for this typhoid fever risk factor analysis on the basis of potential environmental risk factors of interest and potential confounding covariates (*26*). These variables included age, education, self-reported typhoid fever vaccination status, type of toilet at home, type of sewage system, work location, urbanization, and several flooding-related variables (Figure 1; Technical Appendix Table 2).

### Vi-Specific Serologic Analysis

We determined Vi-specific antibody levels by using an ELISA adapted from Rondini et al. (*27*). In brief, we coated ELISA plates with Vi polysaccharide antigen, blocked with nonfat milk buffer, incubated with alkaline phosphatase–conjugated anti-human IgG, and incubated with participant serum samples (dilution 1:200). We detected antibody binding by using *p*-nitrophenyl phosphate substrate (Sigma-Aldrich, St. Louis, MO, USA), and measured absorbance at 405 nm. As reported by Watson et al. (*26*), we used a cutoff of >64 ELISA units for a Vi-seropositive result.

### Geospatial Mapping and Clustering

We estimated the geographic centroid of each community by averaging latitude and longitude coordinates of households sampled within each community and computed typhoid fever seroprevalence for each georeferenced community by using the Vi-seroimmune status of participating persons who resided in each community. We obtained confirmed typhoid fever case incidence data from the Fiji Ministry of Health and mapped per subdivision. All geographic coordinates of communities were displayed in the local projected coordinate system (Fiji Map Grid 1986).

We used Global and Anselin local Moran *I* tests to identify statistically significant spatial clusters and conducted by using GeoDa version 1.6.7 (Technical Appendix) (*28**,**29*). Vi seroprevalence was log-transformed, separate row-standardized spatial weight matrices were calculated on the basis of an inverse-distance relationship, and global and local spatial associations were analyzed within each division.

### Environmental Variables

We downloaded administrative boundaries from the Fiji Global Administrative Divisions Map (*30*). The largest administrative boundaries are known as divisions (i.e., Central, Western, Northern, and Eastern), and the island of Viti Levu is composed of Central and Western Divisions, and the island of Vanua Levu is the Northern Division. Smaller island groups comprise the administrative Eastern Division (in which samples were not collected for this study). Divisions are divided further into 14 subdivisions.

We obtained geospatial environmental data (topography data [elevation and slope], climate data [annual rainfall, rainfall during the wettest month, total rainfall for the cyclone season], hydrology data [rivers and creeks], and soil data [soil type according to composition and drainage quality]) (*31*,*32*) from the University of South Pacific (Suva, Fiji). Euclidean distance maps of straight-line distance to major rivers and creeks were generated from hydrology maps and for poorly drained soils from soil maps. We also provide additional details of spatial data used in the study (Technical Appendix Table 3).

We generated a deterministic flood-risk model based on the principle that depressions and poorly drained soils are more likely to collect rainwater and be flooded (*33*). We also provide additional details on development of this flood-risk map (Technical Appendix Figure 1).

Except for rainfall variables, which we extracted at the community level, we extracted remaining environmental data at the individual geospatially coded household level by using bilinear interpolation. We performed all geospatial processing and mapping by using ArcGIS version 10.2 (Esri, Redlands CA, USA).

### Multilevel Mixed-Effect Logistic Regression

We identified risk factors for Vi antigen seropositive status by using multilevel mixed-effects logistic regression (also known as a generalized linear mixed-effect model) by including environmental and individual-related covariates as fixed-effect and a random intercept. First, we ran a null multilevel mixed-effects logistic model with typhoid fever seroimmune status (binary variable) as the dependent variable. We generated the variance partition coefficient and a caterpillar plot (Technical Appendix Figure 2) by using community residuals.

We tested 16 environmental covariates (Table 1) in the univariable analysis. Regarding continuous independent variables, if analysis showed at least moderate evidence of an association with seropositivity (p<0.05), we then used the variable in multivariable analysis as a continuous variable. However, if analysis showed weak or no evidence of an association with typhoid seropositivity (p>0.05), we then divided the continuous variable into quintiles (Technical Appendix Table 3) that were retested in the univariable model separately as categorical or ordered-categorical variables. We tested all continuous variables associated with Vi antigen seropositivity with p<0.10 for collinearity. We then grouped variables with high collinearity (correlation coefficient >0.8) and included the variable with the smallest p value from each group in multivariable analysis.

**Table 1:** Association between environmental factors and typhoid fever seropositivity by univariable multilevel mixed-effects logistic analysis, Fiji*

Environmental variable	No.	Variable type	Odds ratio (95% CI)	p value
Survey data				
Is there a stream nearby?	1,508	Binary	1.09 (0.82–1.46)	0.528
No (0)	616			
Yes (1)	892			
No. times house has flooded in past 3 y	1,483	Categorical		
0	1,380		1.00 (referent)	NA
1–2	97		0.87 (0.52–1.47)	0.604
3–5	6		0.89 (0.15–5.13)	0.897
No. times land has flooded in past 3 y	1,496	Categorical		
0	1,264		1.00 (referent)	NA
1–2	174		1.13 (0.77–1.66)	0.534
3–5	58		1.21 (0.66–2.22)	0.542
Work location†	1,359	Categorical		
Indoors	636		1.00 (referent)	NA
Outdoors	267		1.59 (1.15–2.19)	0.005‡
Both	456		1.22 (0.93–1.60)	0.160
Urbanization†	1,510	Categorical		
Urban	500		1.00 (referent)	NA
Periurban	247		0.61 (0.37–1.01)	0.054
Rural	763		1.27 (0.89–1.81)	0.185
Geospatial data				
Elevation, by quintiles	1,462	Ordered, categorical	1.02 (0.90–1.15)	0.793
Slope, by quintiles	1,462	Ordered, categorical	1.04 (0.93–1.15)	0.519
Temperature, by quintiles	1,462	Ordered, categorical	0.95 (0.84–1.07)	0.398
Annual rainfall, by quintiles†	1,462	Ordered, categorical	1.13 (1.01–1.28)	0.039‡
Rainfall in wettest month, by quintiles	1,462	Ordered, categorical	1.15 (1.02–1.30)	0.020‡
Rainfall during cyclone season, by quintiles	1,462	Ordered, categorical	1.14 (1.01–1.29)	0.029‡
Distance to major rivers, by quintiles	1,462	Ordered, categorical	1.07 (0.95–1.20)	0.255
Distance to major rivers and major creeks, km†	1,462	Continuous	0.99 (0.99–1.00)	0.081
Distance to major rivers and major and minor creeks, by quintiles	1,462	Ordered, categorical	0.96 (0.86–1.07)	0.439
Distance to poorly drained soils (major and secondary flood plains), by quintiles	1,462	Ordered, categorical	0.92 (0.80–1.06)	0.275
Distance to poorly drained soils (major flood plains only), by quintiles	1,462	Ordered, categorical	1.00 (0.87–1.17)	0.949
Distance from modeled flood-risk area, by quintiles†	1,462	Ordered, categorical	0.90 (0.78–1.03)	0.134

*NA, not applicable. †Variables included in the multivariable multilevel analysis. ‡Variables strongly associated with typhoid fever seroimmune status by univariable analysis (p<0.05).

In addition to 5 environmental variables (Table 1), we confirmed several nonenvironmental risk factors (i.e., age, education, self-reported typhoid fever vaccine status, type of home toilet, type of sewage system, and knowing persons who have had typhoid fever ) for *Salmonella* Typhi Vi antigen seropositivity as significant risk factors by univariable analysis (Technical Appendix Table 2). We included these factors in the multivariable analysis. We developed parsimonious regression models by using a backward stepwise variable selection approach, eliminating 1 variable at a time on the basis of the highest p value in a likelihood ratio test and retaining only variables with p<0.05. We validated the final fitted multivariable statistical model by using the Hosmer-Lemeshow test and by generating predicted typhoid seroprevalence values for sampled communities (Technical Appendix Figure 2). We analyzed data by using Stata version 14 (StataCorp LLC, College Station, TX, USA).

### Boosted Regression Trees Modeling

We developed a base model by using the location of communities (latitude and longitude) and those variables that were found to be associated with Vi antigen seropositivity by univariable analysis. We conducted a simplification of the base model by removing redundant or noninformative variables and used an ensemble of 50 boosted regression trees (BRT) models with 11 of the most influential predictors and random sampling of 1,305 samples (a minimum of 750 sampled at 1 time) to estimate relative contributions and marginal effect plots of the most influential variables (additional details on the BRT model in the Technical Appendix). We conducted BRT modeling in R version 3.2.2 (http://www.R-project.org) by using the gbm package (*34*).

## Results

### Detection of Typhoid Fever Hotspot Communities

Approximately one third of serum samples (485/1,516) were seropositive for Vi-specific antibodies (Technical Appendix Table 1). Vi antigen seroprevalence for sampled communities in Fiji ranged from 8% to 65%; estimates were 35% for the Central Division and 24% for the Western Divisions (Figure 1). Furthermore, although the Northern Division (Vanua Levu) has a smaller population, it had a Vi antigen seroprevalence of ≈40%.

**Figure 2:**
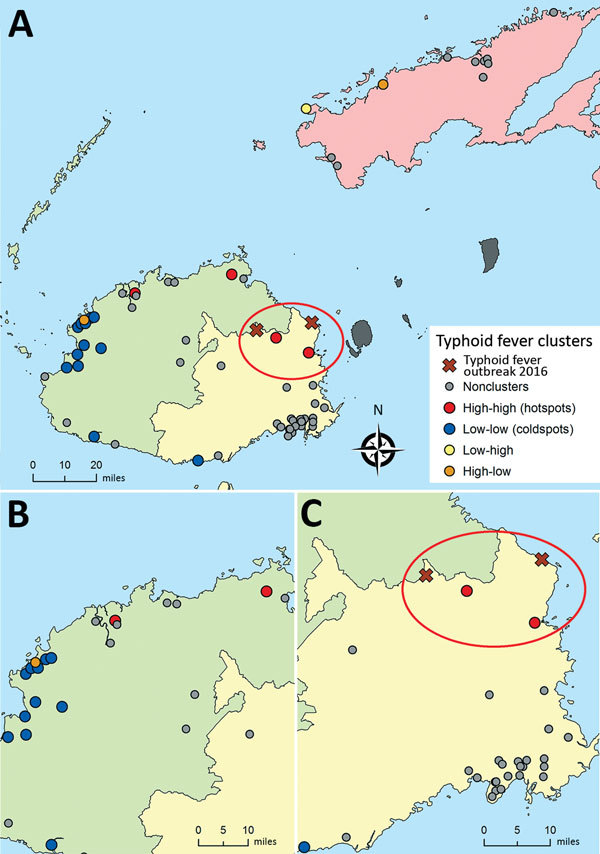
Local clustering of seroprevalence of typhoid fever in divisions in Fiji. Local Anselin Moran I analysis conducted for each division separately by using an inverse-distance weighting for the communities within 3 divisions. A) Northern, B) Western, and C) Central. High-high clusters (hotspots) are communities with high seroprevalence of antibodies against *Salmonella enterica* serovar Typhi Vi capsular antigen that are near other communities with high seroprevalence. Low-low clusters (coldspots) are communities with low seroprevalence of antibodies against *Salmonella* Typhi Vi antigen that are near other communities with low seroprevalence. Red ovals indicate locations of the typhoid outbreak in 2016 after Cyclone Winston and hotspots detected by local clustering.

Global Moran *I* analysis showed strong evidence of geographic clustering of Vi antigen seroprevalence for sampled communities in the Western Division (*I* = 0.49, p = 0.002) and weak evidence for the Central (*I* = 0.08, p = 0.08) and Northern (*I* = −0.42, p = 0.10) Divisions. The Anselin Local Moran *I* test showed that, although Vanua Levu had high typhoid fever seroprevalence, there was no apparent typhoid fever hotspot clustering for communities on this island (Figure 2, panel A). However, 4 high-high (hotspot) seroprevalence cluster communities were detected in the northern and northeast regions of the Western and Central Divisions (Figure 2, panel B, C), whereas coldspots were detected primarily in the Western Division (Figure 2, panel B).

### Multilevel Univariable and Multivariable Analyses

Univariable analysis identified 4 environmental variables (work location, annual rainfall, rainfall during the wettest month, and rainfall during the cyclone season) and 4 nonenvironmental variables (age, education, sewage disposal, typhoid fever vaccination status) as having a significant association with Vi antigen seropositivity (p<0.05) (Table 1; Technical Appendix Table 1). Furthermore, we found suggestive evidence of an association with Vi antigen seropositivity (0.1>p>0.05) for several other environmental and nonenvironmental variables (urbanization, distance to major rivers and major creeks, toilet type, knowing persons who have had typhoid fever) (Table 1; Technical Appendix Table 2).

We included 1 rainfall variable and all other environmental and nonenvironmental factors with at least a suggestive association (p<0.01) in the multivariable multilevel logistic regression analysis (Table 1; Technical Appendix Table 2). We also included proximity to modeled flood-risk areas as a fixed-term in the final fitted multivariate model regardless of its evidence of association on the univariable analysis because other environmental factors (such as rainfall and proximity to rivers) might have confounded the univariable analysis. The final multivariable statistical model contained 6 variables that explained the variation in Vi antigen seropositivity for sampled persons and communities.

After we adjusted for potential confounders (age, typhoid fever vaccination, and flush toilets), we found that annual rainfall showed a positive association (odds ratio [OR] 1.26/quintile increase; p<0.001). We also found that distance to major rivers and major creeks (OR 0.89/km increase; p = 0.031) and to modeled flood-risk areas (OR 0.80/quintile increase; p = 0.002) showed negative associations with Vi antigen seropositivity (Table 2).

**Table 2:** Association between social and environmental factors and typhoid fever seroimmune status in multivariable multilevel model, Fiji*

Variable	Odds ratio (95% CI)	p value
Annual rainfall, by quintiles	1.26 (1.12–1.42)	<0.001
Distance to major rivers and major creeks, km	0.89 (0.80–0.99)	0.031
Distance to modeled flood-risk areas, by quintiles	0.80 (0.69–0.92)	0.002
Age of participant, y	1.03 (1.02–1.03)	<0.001
Vaccination status	1.62 (1.02–2.57)	0.041
Type of toilet at home	NA	NA
Flush	1.0 (referent)	NA
Water seal/pour flush	1.66 (1.16–2.38)	0.006
Pit (with or without slab) and bucket	1.51 (0.91–2.52)	0.110

*Multivariable model was run by using 1,338 observations in 61 communities. NA, not applicable.

The fitted model not only explained fixed-effect variation across persons, but also some of the variation across sampled communities. Comparison of the null and final models showed a reduction in the variance partition coefficient from 7.6% (p<0.0001) to 2.1% (p<0.0001), which indicated that the final statistical model explained 72% of the variation in seropositivity between communities. We validated the final multivariable model fitted by using the Hosmer-Lemeshow test and found that predicted proportions computed for the individual level were not significantly different from those for the observed proportions (p = 0.558) (Technical Appendix Figure 2).

### Boosted Regression Tree Modeling

We estimated that age, GPS location, and the 3 environmental factors (distance to major rivers and creeks, distance to flood-risk areas, and annual rainfall) were the major predictors of Vi antigen seropositivity in Fiji (Table 3). These 6 covariates accounted for ≈90% of the estimated relative contribution to Vi antigen seropositivity.

**Table 3:** Relative contributions of predictor variables from an ensemble of 50 boosted regression tree models for typhoid fever seropositivity developed with cross-validation on data from 1,305 samples and 11 variables, Fiji

Variable	Data type	Relative contribution, % (95% CI)
Age, y	Continuous	33.0 (31.1–34.8)
Longitude, °E	Continuous	15.5 (14.7–16.0)
Distance from major rivers and creeks, m	Continuous	14.5 (13.6–15.3)
Annual rainfall, mm	Continuous	9.3 (8.5–10.0)
Distance from flood-risk areas, m	Continuous	7.7 (6.8–8.4)
Latitude, °S	Continuous	6.9 (5.6–7.9)
Education	Categorical	4.2 (3.8–4.6)
Urbanization	Categorical	3.3 (2.9–3.8)
Typhoid fever vaccination	Binary	2.3 (2.1–2.5)
Sewage disposal	Categorical	1.8 (1.5–2.2)
Toilet type at home	Categorical	0.8 (0.6–1.2)

**Figure 3:**
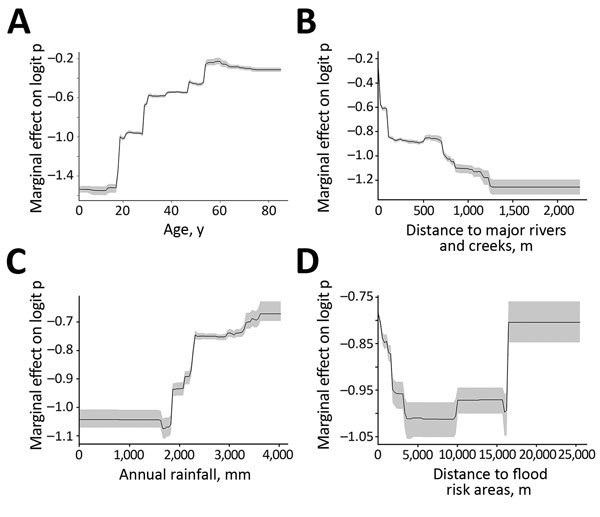
Partial dependence plots for the 4 most influential variables in boosted regression tree (BRT) model for antibodies against Vi capsular antigen of *Salmonella enterica* serovar Typhi, Fiji, 2013. A) Age; B) distance to major rivers and creeks; C) annual rainfall; and D) distance to flood-risk areas. The final ensemble BRT was constructed with 50 BRT models and 11 environmental and social covariates by using data from 1,305 samples. Gray areas indicate 95% CIs of plots.

The marginal effect plot for age showed that most exposure to *Salmonella* Typhi occurred in persons <40 years of age and rates plateaued in persons >60 years of age (Figure 3, panel A). Distances <1,300 m to major rivers and major creeks were predicted to increase Vi antigen seropositivity, with distances <200 m showing the greatest effect (Figure 3, panel B). Annual rainfall had a minimal effect on Vi antigen seropositivity until ≈1,700 mm, above which the risk increased dramatically (Figure 3, panel C). Furthermore, shorter distances to modeled flood-risk areas showed some contribution to typhoid fever seropositivity (Figure 3, panel D).

## Discussion

In the past 2 decades, Fiji has observed a steady increase in confirmed typhoid fever cases (*16*–*18*). However, little is known about the geospatial distribution and underlying risk factors of typhoid fever in Fiji. Our study demonstrated a spatially heterogeneous exposure to typhoid fever across Fiji, with Vanua Levu island showing the highest seroprevalence. High-seroprevalence communities (hotspots) were detected only in Viti Levu, whereas typhoid fever appeared to be more homogeneously distributed in Vanua Levu, suggesting a different transmission pattern on the 2 islands. Annual rainfall and proximity to major rivers, creeks, and potentially floodable areas were major environmental risk factors for serologic evidence of exposure to *Salmonella* Typhi in Fiji.

The Vi antigen seroprevalence distribution closely resembled the typhoid fever case incidence pattern reported by the Fijian Ministry of Health during 2008–2013 (Technical Appendix Figure 3). Vanua Levu and northeastern Viti Levu had the highest typhoid fever burden. In April 2016, after Cyclone Winston hit Fiji, there was a sudden outbreak of typhoid fever in the villages of Qelekuro and Nabulini (*35*), which are located in northeastern Viti Levu. This latest typhoid fever outbreak in Fiji supports our findings of high-risk areas for *Salmonella* Typhi exposure, particularly in northeastern Viti Levu (Figure 2, panel A), and reinforces the hypothesis of increased exposure to typhoid fever caused by environmental anomalies in the aftermath of a cyclone.

Similar to our findings, other studies have found positive associations between diseases transmitted by the fecal–oral route (such as cholera and typhoid) and waterborne diseases (such as leptospirosis) with heavy rainfall and proximity to major rivers (*36*–*40*). Heavy rains in Fiji, particularly during the cyclone season (November–April) (*21*), might lead to overflowing of septic tanks and contamination of the local environment and drinking water sources. Furthermore, our study indicated proximity to major rivers and creeks as a risk factor for acquiring *Salmonella* Typhi, probably because major rivers and creeks are used in Fiji (similar to many other middle-income countries) for washing clothes, taking baths, and swimming (*41*). In addition, streams near populated areas can become contaminated by cyclones or heavy rains that cause overflowing of sewage and waste systems. Therefore, future studies investigating environmental risk factors should sample surrounding water sources for water quality assessment.

Incidences of many foodborne and waterborne diseases have been shown to increase soon after heavy flooding (*22*–*24*,*42*). Fiji had outbreaks of typhoid fever and leptospirosis after devastation and flooding caused by cyclones (*16*,*18*,*40*,*43*). Our multivariate model demonstrated an increased risk for *Salmonella* Typhi infection for persons living closer to the modeled flooding areas. Annual cyclone season and heavy rainfall, combined with most of the population in Fiji living in low-lying coastal areas, make exposure to flooding a common phenomenon and a potential conduit of *Salmonella* Typhi transmission.

A major strength of this study was the unbiased, individual-level assessment of environmental factors specific to each participant on the basis of their residential GPS coordinates. Furthermore, the large sample number analyzed enabled inclusion of a large number of independent variables (major nonenvironmental risk factors and environmental variables) in the statistical modeling. 

However, despite many strengths, the study also had several limitations. Although Vi antigen–specific antibodies were measured as a proxy for *Salmonella* Typhi infection, the exact role and dynamics of Vi antigen–specific antibodies after *Salmonella* Typhi infection remain unclear. For example, antibodies against Vi antigen have been found to be short-lived, more often associated with chronic carriage (*11*,*13*), and produced as a result of typhoid fever vaccination. Furthermore, geospatial cluster analysis was partially hampered by an uneven distribution of surveyed communities. To mitigate this potential spatial bias, we conducted spatial clustering analysis separately for each division.

Our study was an in-depth investigation of the spatial epidemiology of typhoid fever in Fiji and flooding as a risk factor for transmission of *Salmonella* Typhi. Findings of this study can be used to improve future typhoid fever control programs. Recent outbreak detection in high seropositivity areas (*35*) suggests that serosurveillance for IgG against Vi antigen offers potential for identification of areas and communities at higher risk for typhoid fever. This spatial epidemiology analysis suggests flood-prone areas and other communities near major rivers and creeks or in high-rainfall areas could be prioritized for stricter flood control and typhoid fever preventive measures, such as improved sanitation, provision of secure water sources, and typhoid fever vaccination campaigns.

Technical Appendix Additional information on role of environmental factors in shaping spatial distribution of *Salmonella enterica* serovar Typhi, Fiji.
