# Supplementary material for: Role of Environmental Factors in Shaping Spatial Distribution of Salmonella enterica Serovar Typhi, Fiji
Source: Emerg Infect Dis. 2018 Feb;24(2):284–93. doi: 10.3201/eid2402.170704 (PMC5782885; doi:10.3201/eid2402.170704)
Supplement: Technical Appendix — Additional information on role of environmental factors in shaping spatial distribution of Salmonella enterica serovar Typhi, Fiji. [file 17-0704-Techapp-s1.pdf]

Article DOI: https://doi.org/10.3201/eid2402.170704

Role of Environmental Factors in Shaping Spatial Distribution of *Salmonella enterica* Serovar Typhi, Fiji

Technical Appendix

Building a Flood-Risk Model

The flood-risk model was created in 4 main steps. First, we created a map depicting depression sites (or sink areas) by using the digital elevation model (DEM) raster. A convex or depression surface was obtained with the formula; original DEM – mean DEM, where values <0 were identified as convex zones. First, a mean DEM raster was created by averaging the elevation of 10 ×10 neighboring (i.e., a 250 m × 250 m area). A depression map was then obtained by subtracting the mean DEM raster from the original DEM map, and selecting only the regions with negative pixel values. Second, areas selected as potential flooding areas where those that were convex and fall within an elevation range between 0 m and 40 m, which is approximately the elevation range corresponding to the lower alluvial plains, which is generally affected during severe flooding (*1*). Third, a raster map with poorly drained soils was then created by using the polygon features ranging from imperfectly to very poorly drained soils. Fourth, a new raster flood-risk map was created by using only the overlapping regions of the depressions map and the poorly drained soils map. These overlapping regions were marked as regions at high risk for flooding. Finally, a surface map estimating Euclidean distances to these high-risk flooding regions was created.

Implementation of Spatial Autocorrelation Analysis

Global Moran's *I* statistic (*2*) was used to account for the global spatial autocorrelation of typhoid fever seroprevalence. For the Moran's *I* statistic, the sum of covariations between the sites for the distance *d(i,j)* was divided by the overall number of sites $W(d_{i,j})$ within the distance class *d(i,j)*. Thus, the spatial autocorrelation coefficient for a distance class *d(i,j)* was the average value of spatial autocorrelation at that distance.

$$I = \frac{\mathrm{n}}{\mathrm{S}_p} \frac{\sum_{i=1}^{n} \sum_{j=1}^{n} W_{ij}(\gamma_i - \overline{\gamma})(\gamma_j - \overline{\gamma})}{\sum_{i=1}^{n} (\gamma_i - \overline{\gamma})^2}, \text{ where}$$

n = the sample size;

$W_{ij} = \begin{cases} 1 \text{ if sites i, j are neighbours} \\ 0 \text{ otherwise} \end{cases}$ = row-standardized spatial weights matrix of sites *i* and *j;*

$$S_p = \sum_{i=1}^{n} \sum_{j=1}^{n} W_{i.j} = \text{sum of the number of sampling locations per distance class;}$$

$$\gamma_i = \text{the value at community } i; \text{ and } \bar{\gamma} = \text{global mean value}$$

The actual value for Moran's *I* was then compared with the expected value under the assumption of complete randomization.

$$E(I) = -\frac{1}{n-1}$$

Moran's *I* values may range from −1 (disperse) to +1 (clustered). A Moran's *I* value of 0 suggests complete spatial randomness. To verify that the value of Moran's *I* was significantly different from the expected value, a Monte Carlo randomization test was applied with 9,999 permutations to achieve highly significant values. This statistic is a global statistic in that it averages all cross outcomes over the entire domain.

A local version, called the local indicator of spatial association or Anselin Local Moran's *I* statistic (*3*) enabled us to test for statistically significant local spatial clusters, including the type and location of these clusters. It is calculated as

$$I_i(d) = \frac{(\gamma_i - \bar{\gamma})}{\frac{1}{n}\sum_{i=1}^{n}(\gamma_i - \bar{\gamma})} \sum_{i=1}^{n} W_{ij}(d)(\gamma_i - \bar{\gamma}), \text{ where}$$

$W_{ij}(d)$ is the row-standardized weights matrix given a local neighborhood search radius *d.* The conceptualization of spatial relationship (i.e., neighborhood definition) was the same as the global statistics that were applied. Unlike the global Moran's *I*, which has the same expected value for the entire study area, the expected value of local Moran's *I* varies for each sampling location because it is calculated in relation to its particular set of neighbors.

$$E(I_i) = -\frac{1}{n-1} \sum_{j=1}^{n} W_{i,j}$$

The significance of the local Moran's *I* was calculated by using a randomization test on the Z score with 9,999 permutations to achieve highly significant values. Positive spatial autocorrelation occurs when a community with a specific typhoid fever seroprevalence is

surrounded by neighboring communities with similar outcome value (low-low, high-high), thus forming a spatial cluster.

Implementation of Boosted Regression Trees Modeling Approach for Typhoid Fever Seropositivity Data

First, a single boosted regression tree (BRT) model was constructed with individual typhoid fever eroimmune status binary data, cross-validation optimization, and accounting for multiway interactions. As per guidelines of Elith et al. (*4*), the learning rate (lr) and tree complexity (tc) were set according to the number of observations and testing different values on a subset of samples (75%) by using deviance reduction as the measure of success. After several tests, an lr of 0.0025 and a tc of 5 were identified as optimal parameters, thereby enabling the model to account for up to 5 potential interactions and slowing it down enough to get the model converged without over-fitting the data. The base model was constructed including location of communities (longitude and latitude) and the 11 variables found to be associated with typhoid fever seropositivity in univariable logistic regression analysis (Technical Appendix Table 4).

A simplification of the base model was constructed by removing redundant or noninformative variables without compromising the predictive performance of the model. This simplification process (implemented by using the function gbm.simplify) was run within a 10-fold cross-validation procedure, progressively simplifying the model fitted to each fold, and using the average cross-validation error to decide how many variables could be removed from original model without affecting predictive performance. An ensemble BRT (i.e., 50 BRT models) was then run with the simplified model using 5 parallel central processing units to attain 95% CIs in the relative contributions of the variables and the marginal effect plots. Relative contributions of variables to typhoid fever seropositivity were estimated by using the ensemble BRT model. Fitted functions of the ensemble BRT model were visualized by graphing marginal effect curves or partial dependence plots, which demonstrate the effect of each independent variable on the typhoid fever seropositive outcome while all other variables in the model are held constant at its average.

References

1. Townsend PA, Walsh SJ. Modeling floodplain inundation using an integrated GIS with radar and optical remote sensing. Geomorphology. 1998;21:295–312. http://dx.doi.org/10.1016/S0169-555X(97)00069-X

2. Moran PA. Notes on continuous stochastic phenomena. Biometrika. 1950;37:17–23. PubMed http://dx.doi.org/10.1093/biomet/37.1-2.17

3. Anselin L. Local indicators of spatial association—LISA. Geographical Analysis. 1995;27:93–115. http://dx.doi.org/10.1111/j.1538-4632.1995.tb00338.x

4. Elith J, Leathwick JR, Hastie T. A working guide to boosted regression trees. J Anim Ecol. 2008;77:802–13. PubMed http://dx.doi.org/10.1111/j.1365-2656.2008.01390.x

Technical Appendix Table 1. Characteristics of samples collected during first survey and those included in statistical analysis of environmental factors in shaping spatial distribution of *Salmonella enterica* serovar Typhi, Fiji*

Characteristic	Value
Survey sample	
Persons	1,560
Communities	65
IgG against *Salmonella enterica* serovar Typhi Vi antigen	1,531
Persons per community, mean (range)	24 (15–28)
Sample included in analysis†	
Persons	1,516
Communities	63
IgG against *S.* Typhi Vi antigen‡	1,516
Seronegative, <64 EU	1,031
Seropositive, $\geq$64 EU	485
GPS coordinates	1,463
Community cluster area, km^2 (IQR)§	0.04 (0.02–0.13)

*Values are numbers unless indicated otherwise. EU, ELISA units; GPS, global positioning system; IQR, interquartile range.
†Samples from pilot study were not included in the present analysis.
‡Samples with missing IgG titers were excluded from analysis.
§Cluster area of each community was assessed by using sampled household locations of each community.

Technical Appendix Table 2. Univariable analysis of nonenvironmental factors for *Salmonella enterica* serovar Typhi Vi antigen seropositvity, Fiji*

Variable	Variable type	Odds ratio (95% CI)	p value
Age, y†	Continuous	1.03 (1.02–1.03)	<0.001
Education	Categorical	NA	NA
None		1.00 (referent)	NA
Primary		1.47 (0.94–2.30)	0.091
Secondary†		1.71 (1.11–2.64)	0.015
University		1.17 (0.71–1.93)	0.546
Toilet at home	Categorical	NA	NA
Flush		1.00 (referent)	NA
Water seal/pour flush†		1.40 (1.00–1.95)	0.051
Pit (with or without slab) and bucket		1.22 (0.75–1.99)	0.425
Sewage disposal at home	Categorical	NA	NA
Piped sewer system		1.00 (referent)	NA
Septic tank†		0.59 (0.35–0.99)	0.048
Pit latrine†		0.65 (0.43–0.99)	0.043
Elsewhere		0.61 (0.28–1.33)	0.215
Typhoid vaccination status (0 = no, 1 = yes)†	Binary	1.67 (1.07–2.59)	0.023
Do you know persons who have had typhoid fever? (0 = no, 1 = yes)†	Binary	1.56 (0.96–2.54)	0.073

*NA, not applicable.
†These nonenvironmental variables were included in multivariable analysis.

Technical Appendix Table 3. Characteristics used in analysis of environmental factors in shaping spatial distribution of *Salmonella enterica* serovar Typhi, Fiji

Characteristic	Resolution, m	Mean ± SE	Range
Elevation, m	25	41.1 ± 89.3	0–761
Slope, °	25	3.02 ± 3.81	0–25.0
Mean temperature, °C	100	25.1 ± 27.5	0–26.1
Annual rainfall, mm	100	2,490 ± 660	0–4,040
Rainfall in wettest month, mm	100	372 ± 76	0–789
Rainfall during cyclone season, mm	100	1,032 ± 195	0–2,055
Distance to major rivers, km	25	1.21 ± 1.74	0–9.8
Distance to major rivers and major creeks, km	25	0.360 ± 0.343	0–2.250
Distance to major rivers and major and minor creeks, km	25	0.148 ± 0.177	0–1.280
Distance to poorly drained soils (major and secondary floodplains), km	25	0.722 ± 1.710	0–11.250
Distance to poorly drained soils (major floodplains only), km)	25	2.370 ± 3.670	0–17.410
Distance from modeled flood-risk area, km	25	1.890 ± 4.260	0–25.540

Technical Appendix Table 4. Range of each category for continuous variables divided into quintiles for analysis of environmental factors in shaping spatial distribution of *Salmonella enterica* serovar Typhi, Fiji*

Variable	Quintile	Range or value
Elevation, m	Q1	0–7
	Q2	8–15
	Q3	16–19
	Q4	20–39
	Q5	≥40
Slope, °	Q1	0.00
	Q2	0.40–1.21
	Q3	1.28–2.29
	Q4	2.36–4.45
	Q5	≥4.46
Temperature, °C	Q1	0–25.19
	Q2	25.20–25.37
	Q3	25.38–25.64
	Q4	25.65–25.81
	Q5	≥25.82
Annual rainfall, mm	Q1	0–1,909
	Q2	1,910–2,265
	Q3	2,266–2,582
	Q4	2,583–3,104
	Q5	≥3,105
Rainfall in wettest month, mm	Q1	0–338
	Q2	339–360
	Q3	361–379
	Q4	380–408
	Q5	≥409
Rainfall during cyclone season, mm	Q1	0–943
	Q2	944–1,001
	Q3	1,002–1,053
	Q4	1,054–1,125
	Q5	≥1,126
Distance to major rivers, km	Q1	0–0.150
	Q2	0.151–0.459
	Q3	0.460–0.908
	Q4	0.909–1.726
	Q5	≥1.727
Distance to major rivers and major creeks, km	Q1	0–0.090
	Q2	0.091–0.195
	Q3	0.196–0.320
	Q4	0.321–0.506

Variable	Quintile	Range or value
	Q5	≥0.507
Distance to major rivers, major and minor creeks	Q1	0–0.025
	Q2	0.026–0.075
	Q3	0.076–0.111
	Q4	0.112–0.200
	Q5	≥0.201
Distance to poorly drained soils (major and secondary floodplains), km	Q1	NA
	Q2	0–0.044
	Q3	0.045–0.152
	Q4	0.153–0.776
	Q5	≥0.777
Distance to poorly drained soils (major floodplains only), km	Q1	NA
	Q2	0–0.276
	Q3	0.277–1.521
	Q4	1.522–4.310
	Q5	≥4.311
Distance from modeled flood-risk area, km	Q1	NA
	Q2	0–0.127
	Q3	0.128–0.576
	Q4	0.577–1.681
	Q5	≥1.682

*NA, not applicable; Q, quintile.

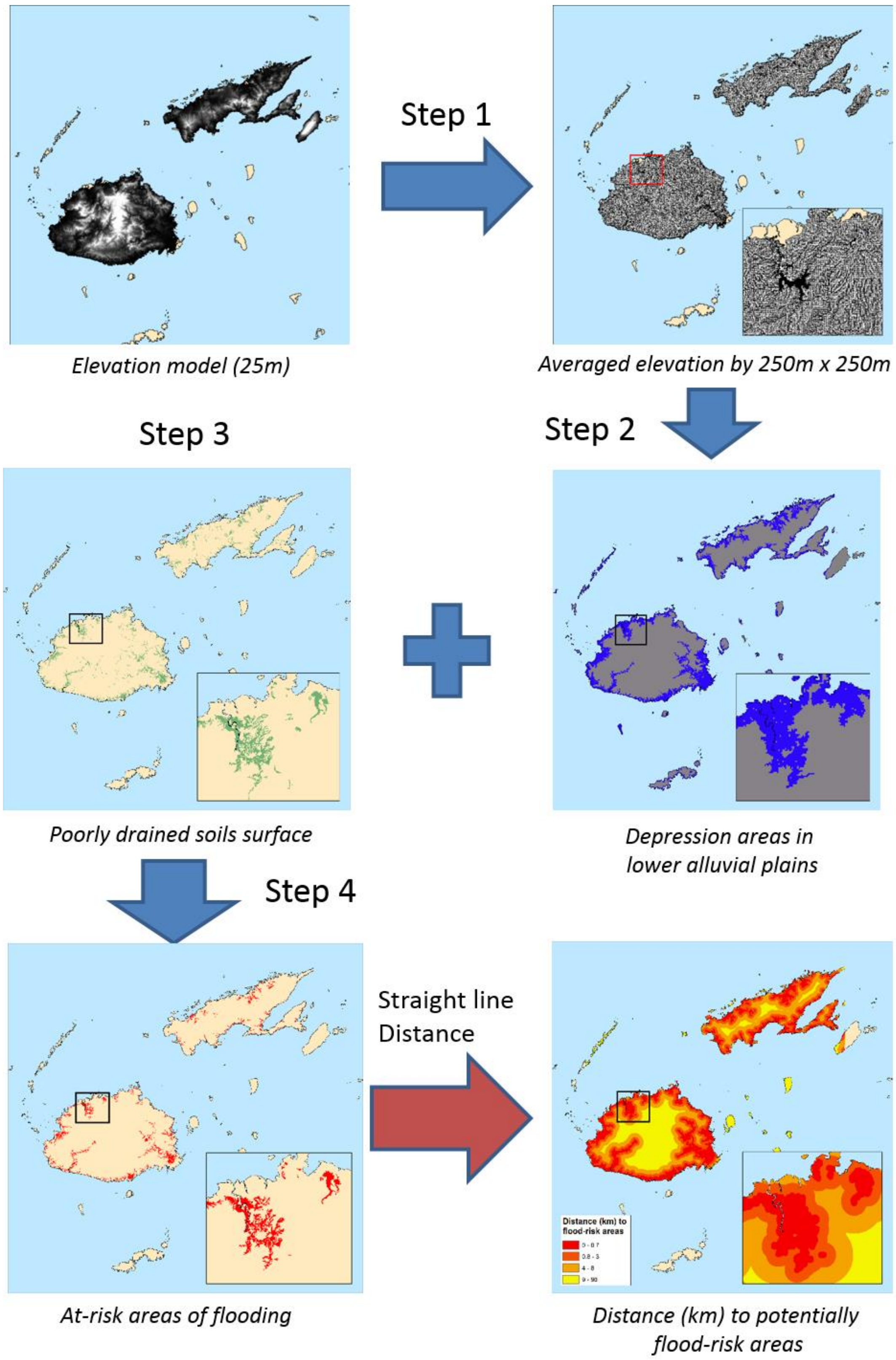

Technical Appendix Figure 1. Development of a flood-risk model for environmental factors in shaping spatial distribution of *Salmonella enterica* serovar Typhi, Fiji. Detailed methods are described in the text.

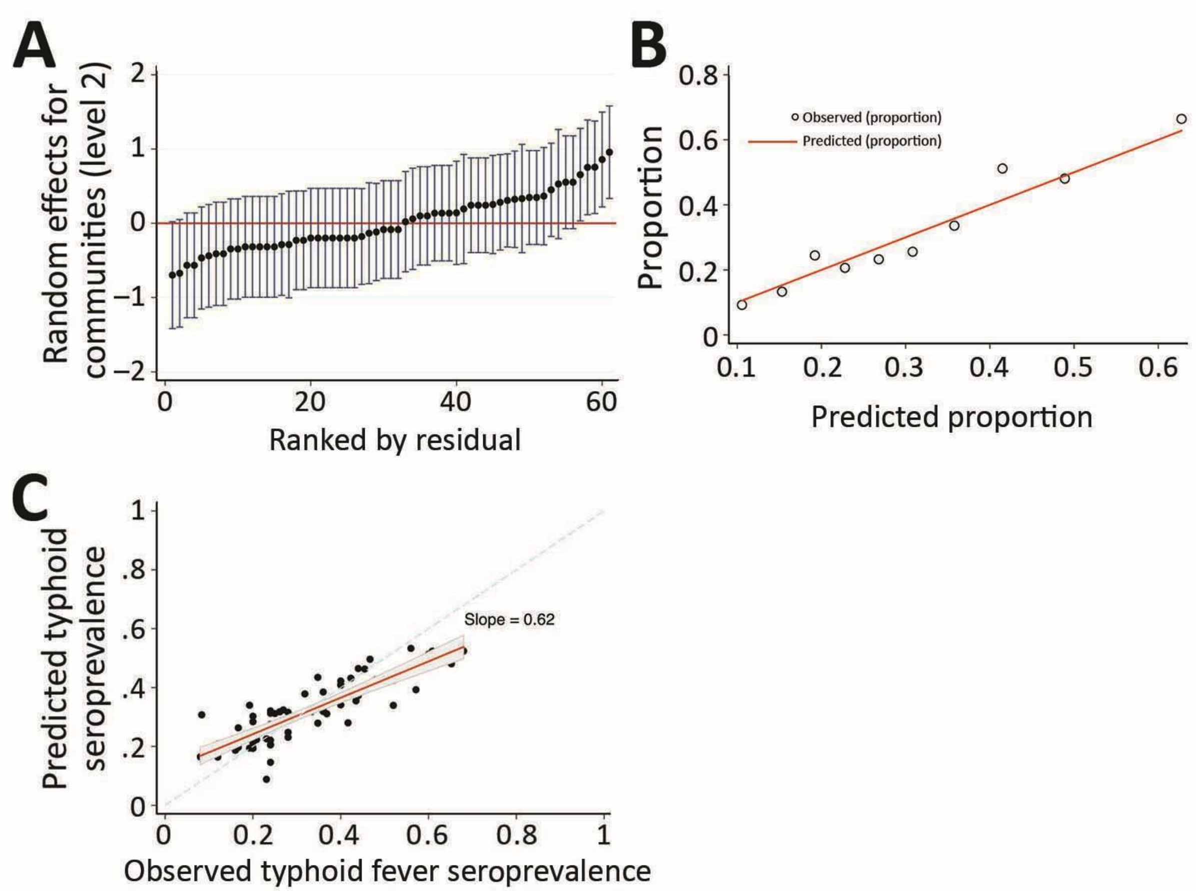

Technical Appendix Figure 2. Validation of the fitted multilevel mixed-effect logistic regression model for environmental factors in shaping spatial distribution of *Salmonella enterica* serovar Typhi, Fiji. A) Distribution of community random effect residuals with 95% CIs to justify the use of a multilevel model. B) Validation of the final multilevel regression model to explain variation in seroimmune status for *Salmonella enterica* serovar Typhi Vi antigen by using the Hosmer-Lemeshow test (p = 0.558) C) Assessing the final statistical model by comparing the predicted and observed typhoid fever seroprevalence at the community level.

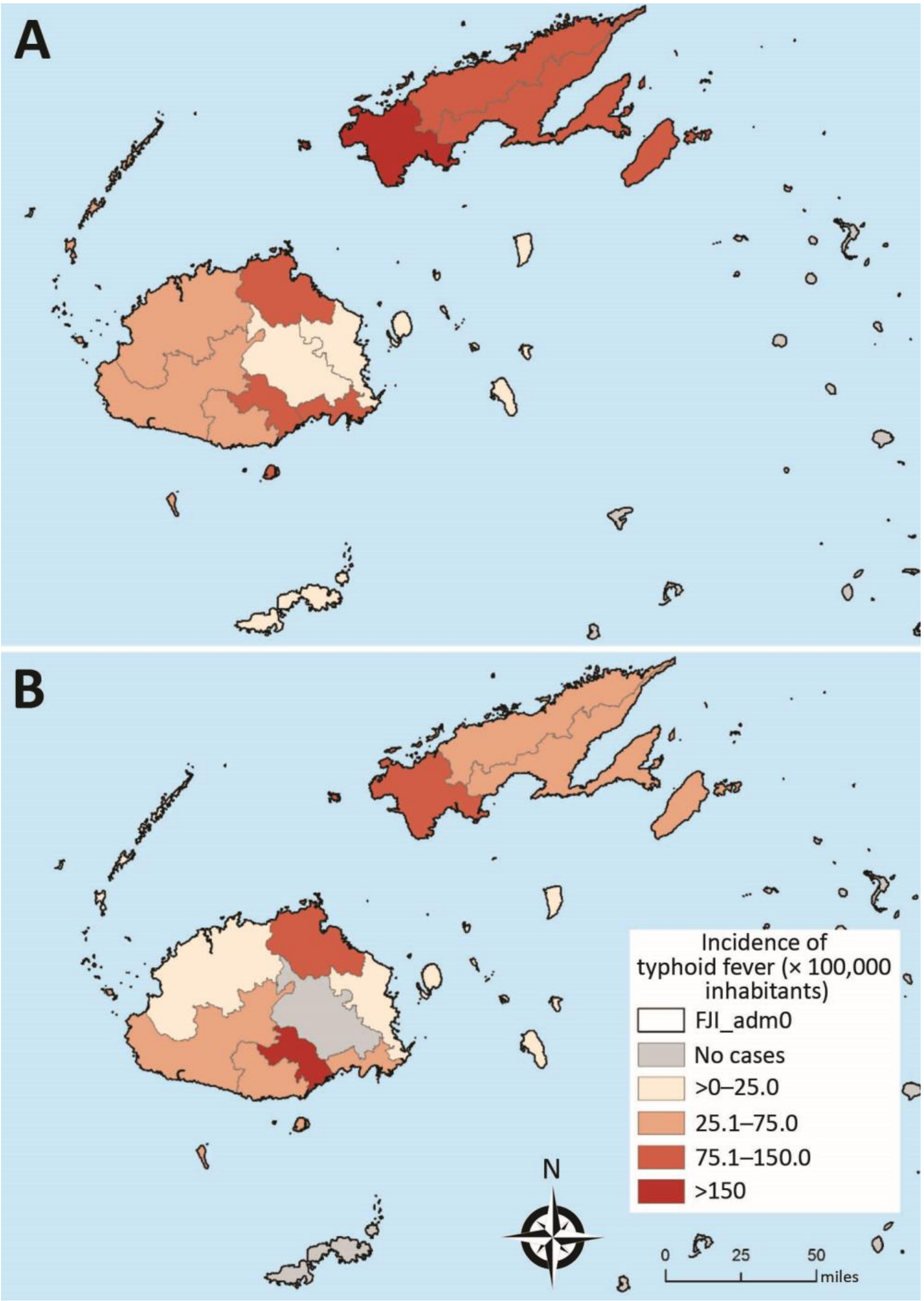

Technical Appendix Figure 3. Confirmed typhoid fever case incidence/100,000 inhabitants reported for each subdivision of Fiji during 2008–2013 and 2014. FJI, Fiji.
